# Mindfulness, Interpretation Bias, and Levels of Anxiety and Depression: Two Mediation Studies

**DOI:** 10.1007/s12671-018-0946-8

**Published:** 2018-04-12

**Authors:** Birgit Mayer, Marike G. Polak, Danielle Remmerswaal

**Affiliations:** Department of Psychology, Education and Child Studies (DEPCS), Erasmus University Rotterdam, P.O. Box 1738, 3000 DR Rotterdam, The Netherlands

**Keywords:** Dispositional mindfulness, Acceptance, Interpretation bias, Information processing, Anxiety symptoms, Depressive symptoms

## Abstract

In two studies, a possible mediation effect was tested of cognitive interpretation bias in the relation between respectively dispositional mindfulness and acceptance, on the one hand, and symptoms of depression and anxiety, on the other hand. An undergraduate student sample (*N* = 133; 86% female, *M*_age_ = 19.8) and a convenience community sample (*N* = 186; 66% female, *M*_age_ = 36.5) were examined by means of an online questionnaire measuring dispositional mindfulness (FFMQ-SF; Study 1) and acceptance (AAQ-II; Study 2), anxiety (STAI-trait) and depressive (BDI-II) symptoms, and interpretation bias (with the interpretation bias task, IBT). Considering both studies, results showed consistently the expected relations of larger mindfulness skills going together with a smaller cognitive interpretation bias and lower levels of depression and anxiety symptoms. More interestingly, it was found that interpretation bias served as a mediator in the relations between respectively dispositional mindfulness and acceptance, and symptoms of depression and anxiety. With these findings, some more insight in the working mechanisms of mindfulness-based treatments on internalizing psychopathology has been obtained.

## Introduction

The mental health field is confronted with rapid scientific support for the new third generation of behavior and cognitive therapies, such as mindfulness-based stress reduction (MBSR; Kabat-Zinn 1990), mindfulness-based cognitive therapy (MBCT; Segal et al. 2001), and acceptance and commitment therapy (ACT; Hayes et al. 1999). Rather than targeting and attempting to alter the content and frequency of cognitions and emotions, as in traditional cognitive therapy models, these mindfulness-based therapies target on altering the relationship with cognitions and emotions. In these therapies, focus is on decentering, which refer to the capacity to shift from one’s subjective experience onto observing that experience, which is put forward as important process for mental health (Bernstein et al. 2015). These therapies appear to be effective for a variety of psychopathology such as chronic pain (Hilton et al. 2016), substance misuse (Li et al. 2017), and depression and anxiety problems (Hofmann et al. 2010).

Traditional cognitive models of psychopathology emphasize the key role of information processing biases in the development and maintenance of various psychological disorders such as anxiety disorders and depression (Beck et al. 1985; Beck and Haigh 2014). In this view, a function of the mechanisms underlying fear is to facilitate detection of danger in the environment and to help the organism respond effectively to threatening situations (see Bar-Haim et al. 2007). This leads to attentional biases, characterized by the finding that attention is drawn to the location of threat stimuli more quickly and easily than to other stimuli (e.g., Fox et al. 2001). In addition, interpretation biases are involved (Beck et al. 1985), which refer to the recurrent interpretation of ambiguous information in a threatening or negative way. Research has indeed demonstrated the significance of biased attention and interpretation in the development and maintenance of psychopathology such as anxiety disorders and depression (e.g., Blackwell and Holmes 2010; De Raedt and Koster 2010). Congruently, reduction in attention and interpretation biases is found to decrease psychopathological symptoms (e.g., Mackintosh et al. 2006; Mathews and Mackintosh 2000).

Among other possible mechanisms of change, mindfulness-based therapies are highly associated with reduced information processing biases (see Hanley et al. 2015). That is, mindfulness is theorized to be linked with more veridical appraisals of experiences and a reduced tendency to evaluate the present moment by previously held expectations and beliefs (Hanley et al. 2015). Mindfulness practice is associated with a “beginner’s mind,” which refers to the ability to observe each experience as something new, without any attention or interpretation bias (Bishop et al. 2004). Likewise, unbiased processing within mindfulness is referred to as accepting and experiencing each moment as it is, not filtered through one’s cognitive apparatus (Lakey et al. 2008). In a similar vein, Brown and Ryan (2004) argued that mindfulness is paying attention carefully to what is actually happening in each moment without trying to alter or change (cf. acceptance), or put a conceptual framework around it. As a final illustration of this idea of unbiased processing, Vago (2014) stated that mindfulness-based practices may operate by replacing the learned maladaptive representations by novel more accurate observations and decreased bias. In other words, it is assumed that through mindfulness practice, one is able to perceive a larger proportion of reality more accurately and that biases related to one’s limited world- and self-view are decreased.

Despite these theoretical implications, so far, relatively little empirical work has focused on the relation between mindfulness practice and basic cognitive processes, which may provide more insight into the mechanisms by which mindfulness-based therapies improve mental health. Some studies, however, indeed found support for the supposed role of mindfulness in reducing cognitive biases. In this context, most studies focused on attentional bias. For example, studies have shown that mindfulness is related to less attentional bias in recovering alcohol dependent patients (Garland et al. 2012) and in formerly depressed adults (De Raedt et al. 2012). Furthermore, in a treatment study, Garland and Howard (2013) provided a first tentative indication that a mindfulness-oriented intervention may reduce attentional bias for pain-related stimuli in chronic pain patients. These studies give a first indication that mindfulness and specific (attentional) information processing are indeed related.

In the information processing domain of negativity bias, the relation of mindfulness was experimentally studied by Kiken and Shook (2011). Most notably, they found that participants who received a mindfulness induction displayed no bias toward negativity and a more accurate observation of positivity. In a cross-sectional study in social phobic participants, Schmertz et al. (2012) found that cognitive appraisals in social situations acted as a mediator in the relation between mindfulness and social anxiety symptoms. In other words, mindfulness related negatively to symptoms of psychopathology (i.e., social anxiety symptoms) via its impact on cognitive (biased) appraisals.

Although mindfulness is usually seen as a state (or mode; Bishop et al. 2004), one could identify people who are more experienced or skilled to practice states of mindfulness than others (Brown et al. 2007). These people could be defined as possessing a higher mindfulness disposition (cf. trait) or everyday mindfulness (Thompson and Waltz 2007). This dispositional mindfulness is also shown to be significantly related to mental health and well-being. For example, Shorey et al. (2014) found in substance use patients that lower trait mindfulness was associated with more severe levels of substance use, depression, and PTSD. Pepping et al. (2016) found in adolescents lower dispositional mindfulness to be related to higher levels of depression, anxiety, and stress. These results fit nicely with the much cited work of Brown and Ryan (2003), in which significant relations are reported between dispositional mindfulness and a wide range of psycho(patho)logical factors, like levels of depressive symptoms, anxiety symptoms, negative affectivity, neuroticism, and, on the positive site, levels of subjective vitality, self-esteem, competence, and life satisfaction.

The above described information processing studies of Kiken and Shook (2011) and Schmertz et al. (2012) seem to suggest that mindfulness is associated with less negative cognitive biases in the domain of interpretation. In two current studies, we aimed to examine this association more explicitly, together with their relations with anxiety and depressive symptomatology. More specifically, it was examined whether interpretation bias would act as a mediator in the negative relation between mindfulness, on the one hand, and levels of anxiety and depression, on the other hand, as this would shed some more light on possible mechanisms of change of the mindfulness-based treatments for anxiety and depressive disorder. In a first study, we investigated these hypothesized relations by measuring participants’ level of dispositional mindfulness as indication of their general tendency to practice states of mindfulness (cf. Brown et al. 2007). In a second study, our aim was to investigate the generalizability of the hypothesized mediation model by broadening the scope to an important element of mindfulness, namely acceptance. Acceptance and mindfulness are inextricably linked concepts, with acceptance of internal experiences being a key-component of mindfulness-based therapies. Likewise, mindfulness is integrated in Acceptance and Commitment Therapy (Hayes et al. 1999, 2012). Both dispositional mindfulness and acceptance can therefore be considered as eminent parts of mindfulness-based practice. Although acceptance is part of most mindfulness definitions (cf. Fletcher and Hayes 2005) and is described as a mechanism of mindfulness (e.g., Gu et al. 2015), acceptance and mindfulness are regarded as separate constructs. Acceptance, or its reverse experiential avoidance, has shown to have incremental validity over mindfulness alone in predicting depression and anxiety (Fledderus et al. 2012). Therefore, in a second study, after investigating dispositional mindfulness as predictor, it was examined whether acceptance as well was related to cognitive interpretation bias, and whether interpretation bias would act as a mediator in the relation between acceptance and levels of anxiety and depression. For both studies, we hypothesized that higher dispositional mindfulness and acceptance relates to a smaller cognitive interpretation bias, and that this interpretation bias serves as a mediator in the assumed negative relation between these mindfulness skills and symptoms of depression and anxiety.

## Study 1

## Method

### Participants

Participants (*N* = 133) were 115 female (86.5%) and 18 male (13.5%) undergraduate students with a mean age of 19.79 years (*SD* = 2.80, range 17–41). By far the largest part had a traditional Dutch background (79.7%), with some participants having other origins, like Turkish (5.3%), Moroccan (2.3%), Antillean (2.3%), Surinam (1.5%), or other (8.9%). The majority (54.1%) did not have any meditation experience, whereas 35.3% had a bit and 10.6% had (quite) some experience with meditation practices.

### Procedure

Data were collected through a web-based survey using the online survey software Qualtrics (Qualtrics, Provo, UT, USA n.d.). Participants were invited to take part in the study using the online university research platform. Interested persons could sign in and consequently received an e-mail with a short introduction and a link to the online questionnaire. Participants were instructed to fill out the questionnaires in one consecutive session without significant sensory disturbances. The questionnaire started with explanation of ethical rules (like anonymous data collection and complete voluntary participation), followed by respectively the IBT, FFMQ-SF, STAI, BDI, and some general questions on demographics factors and meditation experience. In addition, the 20-item PANAS was measured as part of another research project. Subjects received course credits for participation.

### Measures

Dispositional mindfulness was measured with the Dutch short version of the Five Facets Mindfulness Questionnaire (FFMQ-SF; Bohlmeijer et al. 2011). This questionnaire contains 24 items in five subscales (i.e., Observe, Describe, Act with awareness, Nonjudge, Nonreact). Likert response scales range from 1 = *(almost) never true* to 5 = *very often or always true*. Higher total scores indicate higher dispositional mindfulness. Psychometric properties of this scale are good (Bohlmeijer et al. 2011).

Symptoms of depression were measured with the Dutch version of the Beck Depression Inventory (BDI-II; Beck et al. 1996; Van der Does 2002). This 21-item questionnaire consists of four statements per item with increasing symptom severity which are scored from 0 to 3, resulting in a total score of 0–63 with higher scores indicating more depressive symptom severity. The BDI-II has been widely used for research as well as clinical purposes and its psychometric properties have been well established (Van der Does 2002).

Trait anxiety was measured by using the Dutch trait version of the State Trait Anxiety Inventory (STAI; Spielberger et al. 1983; Van der Van der Ploeg et al. 1980). This commonly used scale contains 20 items with response categories ranging from 1 (*almost never*) to 4 (*almost always*), summing up to higher levels of trait anxiety in cases of higher total scores. Psychometric properties of this scale are good (Spielberger et al. 1983).

Interpretation bias was measured with the Interpretation Bias Task (IBT). This task was construed in line with the threatening interpretation bias scenarios from Mayer et al. (2009) and from MacLeod and Cohen (1993). The present task contained 15 short ambiguous scenarios that describe ambiguous scenes with anxiety and depression relevant content; see Table 1 for some examples. Scenarios were directly followed by the possibility to give a free response (“What’s going through your mind?”) to activate one’s own first impression of the ambiguous scene. These primary open responses were not part of the analyses. After that, two pre-set responses (i.e., a negative interpretation and a neutral/slightly positive interpretation) were presented, in counter-balanced order. Participants had to indicate the degree of credibility of each of the two pre-set responses on 100 mm VAS-scales (0 = *not credible at all*, 100 = *highly credible*). A bias index was calculated by subtracting the neutral/positive VAS score from the negative VAS score and subsequently computing the average difference, creating a possible score range of − 100 to 100 with larger positive scores meaning a more negative interpretation of the ambiguous situations (cf. a negative interpretation bias).

**Table 1:** Examples of ambiguous scenarios in the interpretation bias task

Scenario	Pre-set responses
1. You are giving a presentation and notice two persons laughing. What is going through your mind?	They are having fun time together. They are laughing about me.
2. You are about to move to another city. You are wondering how you will like it there. What is going through your mind?	I’m hesitant; maybe I will not get used to this place. It’s going to be fun; there will be a lot to discover.
3. You wake up in the middle of the night because of a loud noise. What is going through your mind?	Probably it’s something outside or at the neighbors. Oh no, there’s an intruder!

### Data Analyses

Data were inspected for normality, linearity, homoscedasticity, and influential cases. Influentiality was assessed with the Cook’s distance greater than 1 criterion (e.g., Field 2013), where none of the cases exceeded the threshold (i.e., max = .17). To test the hypotheses that interpretation bias mediates the negative relationship between dispositional mindfulness and symptoms of depression and anxiety, hierarchical regression analyses were conducted. In separate analyses, we computed a total effect (c), direct effect (c′), and bootstrapped bias-corrected 95% confidence intervals of the indirect effect (ab) using the PROCESS macro in SPSS (Hayes 2012). Confidence intervals that do not include zero indicate a significant indirect effect (i.e., mediation).

## Results

General descriptive statistics are summarized in Table 2. As can be seen, all scales appear to have sufficient to good reliability (.75 < *α*‘s < .94). Furthermore, as expected, the BDI depression scale correlated highly with the STAI trait anxiety scale [*r* = .79, *p* < .001]. Age was not correlated with these questionnaires [all *r*’s < .13, *p*’s > .14; except for the interpretation bias task: *r* = −.20, *p* < .05]. There were also no significant sex differences [*t*(131)‘s < 1.50, *p*’s > .13] other than for the FFMQ mindfulness score [*t*(131) = 2.38, *p* < .05], with a higher mean for men (*M* = 82.61, *SD* = 8.69) than for women (*M* = 77.43, *SD* = 8.58). Also experience to meditation practices was not different for both sexes [*t*(18.96) = 0.26, *p* > .05] and not related to age or any of the questionnaires [all *r*’s < .13, *p*’s > .14].

**Table 2:** General descriptive statistics of questionnaire data

*N* = 133	*α*	*M*	*SD*	Observed range	(Possible range)
FFMQ-SF	.75	78.13	8.75	55–106	(24–120)
BDI-II	.87	9.06	7.47	0–33	(0–63)
STAI-trait	.94	42.52	10.90	23–73	(20–80)
IBT	.78	− 22.66	23.90	− 83.47 to 71.73	(− 100 to 100)

*Note*: FFMQ-SF = Five Facet Mindfulness Questionnaire–short form; BDI-II = Beck Depression Inventory; STAI = State Trait Anxiety Inventory; IBT = Interpretation Bias Task

**Fig. 1:**
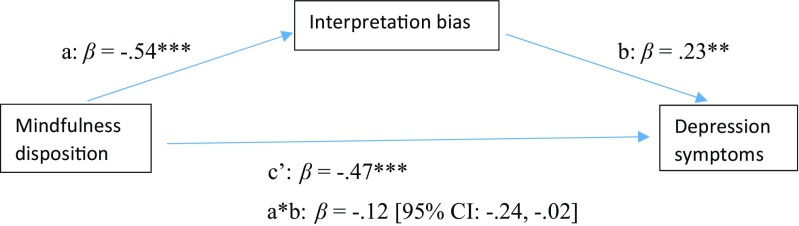
Model displaying *standardized* effects (*β*) between dispositional mindfulness (FFMQ-SF), interpretation bias (IBT) and depression symptoms (BDI-II). Note: The confidence interval for the indirect effect is a BCa bootstrapped CI based on 1000 samples. **p < .01, ***p < .001

In the first mediation model, it was examined whether interpretation bias acted as a mediator in the negative relationship between dispositional mindfulness and symptoms of depression. Outcomes of the analysis in terms of both standardized and unstandardized coefficients are displayed in Fig. 1 and Table 3, respectively. Together, dispositional mindfulness and interpretation bias accounted for 39% of the variance in depression symptoms [*F*(2, 130) = 40.83, *p* < .001]. There was a significant indirect effect (i.e., mediation) of mindfulness disposition on depression symptoms through interpretation bias [a*b: *B* = − 0.10, *SE* = 0.05, 95% BCa CI: − 0.21, − 0.01]. The analysis further indicated that there was a significant total effect of elevated mindfulness disposition inversely predicting level of depression symptoms [c: *B* = −0.51, *SE* = 0.06, *p* < .001]. Dispositional mindfulness was also a significant negative predictor of interpretation bias [a: *B* = − 1.47, *SE* = 0.20, *p* < .001], while interpretation bias significantly predicted level of depression symptoms [b: *B* = 0.07, *SE* = 0.03, *p* < .01]. Finally, when both mindfulness disposition and interpretation bias were included in the mediation model, the direct effect of mindfulness disposition on depression symptoms decreased, but remained significant [c’: *B* = −0.40, *SE* = 0.07, *p* < .001].

**Table 3:** Model estimates (*unstandardized* coef, B) for mediation models in study 1

		Direct effects				Indirect effects		
	*R* ^2^	Path	Coef	SE	*p*	Coeff a*b	SE	95% CI
FFMQ-SF-> IBT->BDI-II	.39^***^	a	− 1.47	0.20	<.001	− 0.10	0.05	− 0.21, − 0.01
		b	0.07	0.03	<.01			
		c	− 0.51	0.06	<.001			
		c’	− 0.40	0.07	<.001			
FFMQ-SF->IBT->STAI	.55^***^	a	− 1.47	0.20	<.001	− 0.22	0.05	− 0.32, − 0.13
		b	0.15	0.03	<.001			
		c	− 0.85	0.08	<.001			
		c’	− 0.63	0.09	<.001			

*Note*. FFMQ-SF = Five Facet Mindfulness Questionnaire – short form; BDI-II = Beck Depression Inventory; STAI = State Trait Anxiety Inventory; IBT = Interpretation Bias Task; The confidence interval for the indirect effect is a BCa bootstrapped CI based on 1000 samples; ***p < .001

**Fig. 2:**
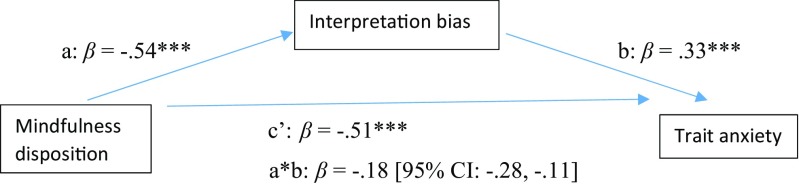
Model displaying standardized effects (*β*) between dispositional mindfulness (FFMQ-SF), interpretation bias (IBT) and trait anxiety (STAI). Note: The confidence interval for the indirect effect is a BCa bootstrapped CI based on 1000 samples. ***p < .001

The second mediation model we tested involved examining the mediation of interpretation bias in the relation between dispositional mindfulness and trait anxiety levels. Outcomes of this analysis are displayed in Fig. 2 and Table 3. Dispositional mindfulness and interpretation bias were found to account for 55% of the variance in levels of trait anxiety [*F*(2, 130) = 78.72, *p* < .001]. There was a significant indirect effect (i.e., mediation) of dispositional mindfulness on trait anxiety through interpretation bias [a*b: *B* = −0.22, *SE* = 0.05, 95% BCa CI: −0.32, −0.13]. The analysis further indicated a significant total effect of mindfulness disposition predicting level of trait anxiety [c: *B* = −0.85, *SE* = 0.08, *p* < .001]. Furthermore, dispositional mindfulness was a significant predictor of interpretation bias [a: *B* = −1.47, *SE* = 0.20, *p* < .001], while interpretation bias was a significant predictor of level of trait anxiety [b: *B* = 0.15, *SE* = 0.03, *p* < .001]. Finally, when both mindfulness disposition and interpretation bias were included in the mediation model, the direct effect of mindfulness disposition on trait anxiety decreased, but remained significant [c’: *B* = −0.63, *SE* = 0.09, *p* < .001].

### Additional Analyses

Since the psychometric support for the FFMQ-SF as a unidimensional measure of mindfulness is somewhat ambivalent (see Bohlmeijer et al. 2011), we conducted additional correlational analyses for the FFMQ-subscales and the other variables in both models of Study 1. Firstly, we found that all FFMQ-subscales correlated significantly with the FFMQ-total score, with *r* ranging from .28 for the Observe-subscale to .66 for the Nonreact-subscale. However, in contrast to the other subscales, which were all significantly inter-related, the Observe-subscale correlated significantly with only one of the other subscales (i.e., Nonreact, *r* = .22, *p* < .01). Furthermore, the Observe-subscale was not significantly correlated with either one of the dependent variables or the mediator. In contrast, the remaining subscales all correlated significantly (*p* < .001) with trait anxiety, depression symptoms, and interpretation bias, with *r* ranging from −.30 to −.68. Finally, we conducted re-analyses of the mediation models with either symptoms of anxiety or depression as the outcome, interpretation bias as the mediator and, in separate analyses, each of the FFMQ-subscales as the predictor. In all cases, the separate FFMQ-subscales showed significant indirect effects, with the exception of the Observe-subscale. Thus, the mediation of interpretation bias in the relation between dispositional mindfulness and either levels of anxiety or depressive symptoms, also exists for the separate subscales of the FFMQ, with the exception of the Observe-subscale.

## Study 2

As stated before, in the second study we examined the generalizability of the Study 1 findings by including acceptance as an important mindfulness-related predictor in the hypothesized model, and by testing a community convenience sample with relatively broad ranges of age and educational level. It was hypothesized that cognitive interpretation bias acts as a mediator in the specific relation between acceptance, on the one hand, and depression and anxiety symptomatology, on the other hand.

## Method

### Participants

Participants were approached via social media (i.e., Facebook), flyers, and e-mail. Participants (*N* = 186) were 122 women (65.6%) and 64 men (34.4%) with a mean age of 36.48 years (*SD* = 14.34, range 18–76). Highest accomplished education in this sample was as follows: 0.5% primary school, 31.7% secondary school (medium level), 7.5% secondary school (high level), 31.7% higher professional education, 16.7% academic level, and 3.2% different from the aforementioned. By far the largest part had a traditional Dutch background (96.2%), with some participants having other origins, like Surinam (2.2%), Turkish (0.5%), or other (1.1%). The majority of this group (56.5%) did not have any meditation experience, whereas 28% had a bit and 15.5% had (quite) some experience with meditation practices.

### Procedure

Similar to the procedure in Study 1, data were collected through a web-based survey using the online survey software Qualtrics (Qualtrics, Provo, UT, USA). Participants were invited to take part in the study using social media and flyers. Interested persons could respond to research assistants and consequently received an e-mail with short introduction and link to the online questionnaire. Participants were instructed to fill out the questionnaire in one consecutive session without significant sensory disturbances. The questionnaire started with explanation of ethical rules (like anonymous data collection and complete voluntary participation), followed by respectively the AAQ-II, IBT, BDI-II, STAI-trait, and some general questions on demographics, treatment history, and meditation experience. Subjects did not receive credits for participation.

### Measures

Similar assessment instruments were used to assess symptoms of depression (BDI-II; Beck et al. 1996; Van der Does 2002), trait anxiety (STAI; Spielberger et al. 1983; Van der Van der Ploeg et al. 1980), and interpretation bias (IBT) as in Study 1.

With the Dutch version of the Acceptance and Action Questionnaire (AAQ-II; Bond et al. 2011; Jacobs et al. 2008), acceptance, as the reverse of experiential avoidance, was measured. The AAQ-II is the default measure of Acceptance and Commitment Therapy (ACT), which is one of the important mindfulness-based third generation cognitive behavior therapies. The AAQ-II was developed from an item pool generated by ACT researchers and therapists (Bond et al. 2011) and is the current form for measuring acceptance (cf. Fledderus et al. 2012). Participants had to rate to what extend they agreed with each of 10 items on a 7-point scale ranging from 1 (*not at all/never true*) to 7 (*very/always true*). A sample item is “I am afraid of my feelings”. After recoding, higher scores indicate higher levels of acceptance. Psychometric qualities are good (Bond et al. 2011).

### Data Analyses

Similar techniques of data inspection and analyses as performed in Study 1, were carried out.

## Results

General questionnaire statistics are summarized in Table 4. As can be seen, all scales appear to have good reliability (all *α*‘s > .78). Furthermore, as indicated by a negative mean interpretation bias score (IBT), participants showed overall a healthy bias favoring benign interpretations to the ambiguous scenarios. As expected, the symptomatology scales BDI-II and STAI were highly correlated [*r* = .81, *p* < .001]. Significant gender differences were found only for BDI-II-scores [*t*(176.08) = 1.98, *p* < .05], with higher levels of depression symptoms for women (*M* = 7.17; *SD* = 6.38) than for men (*M* = 5.64; *SD* = 4.12). Age was not significantly related to any of these scales [all *r*‘s < |.06|; all *p*‘s > .47]. In other words, with regard to acceptance and interpretation bias no differences for gender or relations with age were found. Meditation experience was found to be positively related with age [*r* = .19, *p* = .01] but not with any of the other questionnaire measures. Also no gender differences were found with regard to experience with meditation practice: *t*(184) = 1.66, *p* > .05.

**Table 4:** General descriptive statistics of questionnaire data

*N* = 186	*α*	*M*	*SD*	Observed range	(Possible range)
AAQ-II	.85	52.51	8.57	29–70	(10–70)
BDI-II	.83	6.65	5.74	0–31	(0–63)
STAI-trait	.94	35.74	10.59	20–69	(20–80)
IBT	.78	− 39.93	23.12	− 95.20 to 26.93	(− 100 to 100)

*Note*: AAQ-II = Acceptance and Action Questionnaire; BDI-II = Beck Depression Inventory; STAI = State Trait Anxiety Inventory; IBT = Interpretation Bias Task

**Fig. 3:**
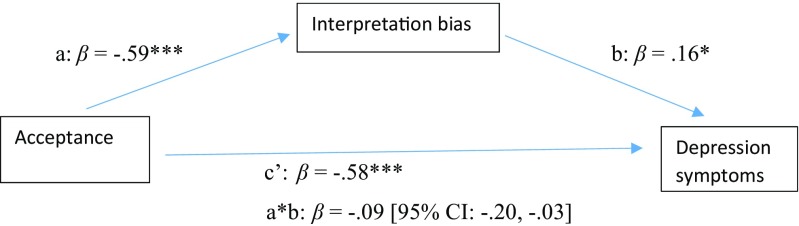
Model displaying standardized effects (*β*) between acceptance (AAQ-II), interpretation bias (IBT) and depression symptoms (BDI-II). Note: The confidence interval for the indirect effect is a BCa bootstrapped CI based on 1000 samples. *p < .05, **p < .01, ***p < .001

Outcomes of the analysis examining the mediation of acceptance and depression symptoms through reduction in interpretation bias reporting both standardized and unstandardized coefficients, are displayed in Fig. 3 and Table 5, respectively. Together, acceptance and interpretation bias accounted for 47% of the variance in depression symptoms [*F*(2,183) = 82.14, *p* < .001]. There was a significant indirect effect (i.e., mediation) of acceptance on depression symptoms through interpretation bias [a*b: *B* = −0.06, *SE* = 0.03, 95% BCa CI: −0.13, −0.01]. The analysis further indicated that there was a significant total effect of elevated acceptance levels inversely predicting depression symptoms [c: *B* = −0.45, *SE* = .04, *p* < .001]. Furthermore, acceptance was a significant predictor of reduced interpretation bias [a: *B* = −1.58, *SE* = 0.16, *p* < .001], while interpretation bias was a significant predictor of increased levels of depression [b: *B* = 0.04, *SE* = 0.02, *p* < .05]. Finally, when both acceptance and interpretation bias were included in the mediation model, the direct effect of acceptance on depression symptoms decreased, but remained significant [c’: *B* = −0.39, *SE* = 0.04, *p* < .001].

**Table 5:** Model estimates (*unstandardized* coef, B) for mediation models in study 2

		Direct effects				Indirect effects		
	*R* ^2^	Path	Coef	SE	*p*	Coef a*b	SE	95% CI
AAQ-II->IBT->BDI-II	.47^***^	a	− 1.58	0.16	<.001	− 0.06	0.03	− 0.13, − 0.01
		b	0.04	0.02	<.05			
		c	− 0.45	0.04	<.001			
		c’	− 0.39	0.04	<.001			
AAQ-II->IBT->STAI	.66^***^	a	− 1.58	0.16	<.001	− 0.13	0.04	− 0.22, − 0.04
		b	0.08	0.02	<.01			
		c	− 0.99	0.05	<.001			
		c’	− 0.86	0.07	<.001			

*Note*. AAQ-II = Acceptance and Action Questionnaire; BDI-II = Beck Depression Inventory; STAI = State Trait Anxiety Inventory; IBT = Interpretation Bias Task; The confidence interval for the indirect effect is a BCa bootstrapped CI based on 1000 samples; ***p < .001

**Fig. 4:**
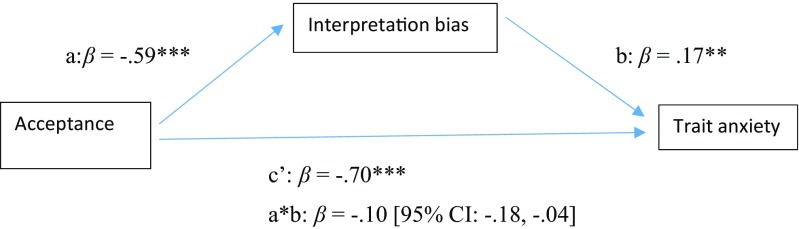
Model displaying standardized effects (*β*) between acceptance (AAQ-II), interpretation bias (IBT) and trait anxiety (STAI). Note: The confidence interval for the indirect effect is a BCa bootstrapped CI based on 1000 samples. **p < .01, ***p < .001

Analyses also supported the second mediation model where we examined whether interpretation bias mediated the association between acceptance and trait anxiety level; see Fig. 4 and Table 5. Together, acceptance and interpretation bias accounted for 66% of the variance in trait anxiety [*F*(2,183) = 174.33, *p* < .001]. There was a significant indirect effect (i.e., mediation) of acceptance on trait anxiety through interpretation bias [a*b: *B* = −0.13, *SE* = 0.04, 95% CI: − 0.22, − 0.04]. The analysis further indicated that there was a significant total effect of elevated acceptance levels inversely predicting trait anxiety level [c: *B* = −0.99, *SE* = 0.05, *p* < .001]. As indicated before, acceptance was a significant predictor of reduced levels of interpretation bias [a: *B* = −1.58, *SE* = 0.16, *p* < .001], while interpretation bias was found to significantly predict levels of trait anxiety [b: *B* = 0.08, *SE* = 0.02, *p* < .01]. Finally, when both acceptance and interpretation bias were included in the mediation model, the direct effect of acceptance on trait anxiety decreased, but remained significant [c’: *B* = −0.86, *SE* = 0.07, *p* < .001].

## Discussion

In these current studies, an attempt was made to investigate the theorized relation between mindfulness and cognitive interpretation bias as a possible mechanism of change in the relation between mindfulness skills and lower levels of anxiety and depressive symptomatology. As mindfulness is theoretically suggested to go along with, or is even defined by, unbiased processing and a beginner’s mind (e.g., Bishop et al. 2004), paying attention to what is actually happening without trying to alter or change (cf. acceptance) (Brown and Ryan 2004), no filtering through one’s cognitive apparatus (Lakey et al. 2008) and veridical appraisals instead of evaluations by expectations and beliefs (Vago 2014), one would expect more mindfully skilled individuals to demonstrate less cognitive interpretation bias when being confronted with ambiguous situations. This association was examined in the present studies by looking at levels of dispositional mindfulness (measured with the FFMQ-sf; Bohlmeijer et al. 2011) and levels of acceptance (measured with the AAQ-II; Bond et al. 2011) as an important element of mindfulness, in respectively a sample of undergraduate students and a community convenience sample in relation to their interpretation bias. Moreover, it was studied whether interpretation bias served as a mediator in the relation between these mindfulness skills, on the one hand, and levels of depression and anxiety, on the other hand, as this would shed some more light on the working mechanisms of mindfulness-based protocols as effective treatments of depression and anxiety problems.

To begin with, results of the present studies support the idea that there are clear relations between dispositional mindfulness and acceptance, and levels of depression and anxiety. In other words, individuals with more (intensive) moments of mindfulness practice during daily life demonstrate less depression and anxiety symptomatology. This is perfectly in line with the third CBT-generation treatment studies showing the effects of mindfulness and acceptance training on several forms of internalizing and externalizing psychopathology (e.g., Hilton et al. 2016; Hofmann et al. 2010; Li et al. 2017).

More interestingly however, results further showed that there is indeed a relation between these mindfulness skills, and cognitive interpretation bias. That is, both in a student sample and a community sample, higher levels of, respectively, dispositional mindfulness and acceptance were found to be substantially related to a smaller amount of negative interpretations of ambiguous daily-life situations. Moreover, this interpretation bias was found to serve as mediator in the relation between dispositional mindfulness and acceptance, on the one hand, and depression and anxiety symptomatology, on the other hand. Naturally, our findings are cross-sectional and thus conclusions of causality and directionality are not possible, since the temporal order of the relations between variables was not established. Consequently, an alternative explanation of the current findings might be that less interpretation bias is related to more dispositional mindfulness and acceptance, which in turn is related to less depression and anxiety. A strategy to investigate which model is more likely that has appeared in the literature, is to compare the size and/or significance of the indirect effect in alternative variants of the model. However, several recent publications, such as Thoemmes (2015) and Lemmer and Gollwitzer (2017), showed that it is not possible to determine merely on a statistical basis which of the different models we could define within the same set of three variables (a so-called equivalence class) is the most plausible. Therefore, further research is warranted to investigate causal directionality. For instance, in an experimental design the direct effect of both mindfulness skills and cognitive interpretation bias on depression and anxiety and on the possible mediators could be compared between groups by using targeted interventions, such as mindfulness-based stress reduction (Kabat-Zinn 1990) and cognitive bias modification (see Hallion and Ruscio 2011; Mathews and Mackintosh 2000).

Nevertheless, we believe that this study makes an important contribution to the understanding of possible mechanisms of the functioning of mindfulness and acceptance on depression and anxiety. The current findings could explain the observed therapeutic effects of mindfulness-based treatments for depression and anxiety problems as being partially obtained by a reduction of interpretation bias. This of course fits nicely with the cognitive models of psychopathology (Beck 1976) and with the mechanism of change approach of cognitive bias modification (CBM-I; e.g., Mathews and Mackintosh 2000). With this CBM-technique, participants are trained away from their biased negative interpretation of ambiguity to adopt a more neutral or positive interpretation. Results of these particular studies indeed showed that reducing interpretation bias resulted in reduced anxiety symptoms (Beard and Amir 2008) and depression levels (Blackwell and Holmes 2010; Lang et al. 2012). Since in CBM-techniques the aim is changing the content of thoughts while in mindfulness-based treatments the key process is changing the attitude to thoughts, it would be interesting to examine in future studies the precise processes of these techniques in relation to interpretation—and other cognitive-bias(es) more in depth and whether they have different effects on generalizability and long-term duration of the effects.

## Limitations

In addition to the cross-sectional nature of the current studies, some other limitations can be defined. First, we tested the hypotheses in two non-clinical samples. Consequently, in both studies the symptomatology measures (i.e., BDI-II and STAI-trait) are on the lower end of the scale and the dispositional mindfulness and acceptance measures (i.e., FFMQ and AAQ-II, respectively) are on the higher end of the scale. Thus, participants were relatively low on psychopathology and relatively high on dispositional mindfulness and acceptance. Therefore, it should be noted that only a limited range of these constructs have been tested. In addition to this, it is relevant to acknowledge that in both studies the mean interpretation bias scores indicated an overall healthy bias favoring benign interpretations to the ambiguous scenarios. In other words, although on a positive note it is worth mentioning that we found in both the student sample and the convenience community sample a comparable strength of significant relations between all these relevant variables, it can of course not be ruled out that these relations differ from those in individuals with clinically high levels of anxiety or depression, an actual negative interpretation bias, and/or considerable low levels of dispositional mindfulness and acceptance. It would be worthwhile to investigate this explicitly in future studies.

As a second additional limitation, in the first study we examined the relations in a student population, with a consequently relatively small range in (young) age and (high) educational level. Moreover, although not related to dispositional mindfulness score, in this (Psychology) student sample a relatively large proportion (i.e., 45.9%) of participants reported having at least a bit of experience with meditation practice. These of course affect the generalizability of the results to the general population. Note, however, that in the second study, a more general public was examined in our community sample. Further, all measures were self-report measures, which makes it susceptible to socially desirable answers or poor introspective capacity. Another limitation of self-report measures is of course common method variance, which may have exaggerated the size of the relational effects. Finally, we measured mindfulness and acceptance as a dispositional trait while it could be argued that mindfulness is typically seen as a state. Although mindfulness is indeed usually defined as a state (or mode; Bishop et al. 2004), one could identify people who are more experienced or skilled to practice states of mindfulness and acceptance, than others (cf. Brown and Ryan 2004). Therefore, individuals in the general population (most of whom have no formal meditation experience) differ reliably in the propensity to be mindful and accepting.

The finding that dispositional mindfulness and acceptance seem to be related to symptoms of depression and anxiety via a reduced cognitive interpretation bias gives us more insight in the possible mechanisms of change of mindfulness-based treatments in the context of depression and anxiety problems. A strong point of the current studies was that the effect of mindfulness was demonstrated in different samples and measuring different features of mindfulness practice. That is, both dispositional mindfulness and the mindfulness-related construct of acceptance were found to partially relate to symptoms of depression and anxiety via reduced interpretation bias. Furthermore, this pertinent mediation relation was obtained in a student sample as well as in a community sample with a broader range of age and educational level.

In future studies, it might be interesting to combine mindfulness disposition, as measured with the FFMQ, and acceptance, as measured with the AAQ-II, together as predictors in the relation with cognitive bias and levels of depression and anxiety, as this would give information how exactly these mindfulness concepts are related in the particular context of information processing biases and psychopathological symptoms. Furthermore, future studies might examine causality of the observed mediation relation by using an experimental set-up where state mindfulness or acceptance is manipulated and negative interpretation bias and internalizing symptoms are measured.

To conclude, our findings indicate that individual differences in mindfulness and interpretation bias relate to individual differences in both symptoms of depression and anxiety. Specifically, the larger the dispositional mindfulness and acceptance and the smaller the cognitive interpretation bias, the lower the levels of depression and anxiety symptoms tended to be. More interestingly, we found that the negative relation between these mindfulness skills and psychopathological symptoms was indirect, accounted for by their shared relation with interpretation bias. In other words, interpretation bias served as a mediator between dispositional mindfulness and acceptance, and symptoms of depression and anxiety. With this finding, some more insight in the working mechanisms of mindfulness-based treatments on internalizing psychopathology has been obtained.

## References

[CR1] Bar-Haim Y, Lamy D, Pergamin L, Bakermans-Kranenburg MJ, Van Ijzendoorn MH (2007). Threat-related attentional bias in anxious and non-anxious individials: a meta-analytic study. Psychological Bulletin.

[CR2] Beard C, Amir N (2008). A multi-session interpretation modification program: changes in interpretation and social anxiety symptoms. Behaviour Research and Therapy.

[CR3] Beck AT (1976). Cognitive therapy and the emotional disorders.

[CR4] Beck AT, Haigh EAP (2014). Advances in cognitive theory and therapy: the generic cognitive model. Annual Review of Clinical Psychology.

[CR5] Beck AT, Emery G, Greenberg RL (1985). Anxiety disorders and phobias: a cognitive perspective.

[CR6] Beck AT, Steer RA, Brown GK (1996). Manual for the Beck depression inventory-II.

[CR7] Bernstein A, Hadash Y, Lichtash Y, Tanay G, Shepherd K, Fresco DM (2015). Decentering and related constructs. A critical review and metacognitive processes model. Perspectives on Psychological Science.

[CR8] Bishop SR, Lau M, Shapiro S, Carlson L, Anderson ND, Carmody J (2004). Mindfulness: a proposed operational definition. Clinical Psychology: Science and Practice.

[CR9] Blackwell SE, Holmes EA (2010). Modifying interpretation and imagination in clinical depression: a single case series using cognitive bias modification. Applied Cognitive Psychology.

[CR10] Bohlmeijer E, Klooster PM, Fledderus M, Veehof M, Baer R (2011). Psychometric properties of the five facet mindfulness questionnaire in depressed adults and development of a short form. Assessment.

[CR11] Bond FW, Hayes SC, Baer RA, Carpenter KC, Guenole N, Orcutt HK (2011). Preliminary psychometric properties of the acceptance and action questionnaire—II: a revised measure of psychological flexibility and acceptance. Behavior Therapy.

[CR12] Brown KW, Ryan RM (2003). The benefits of being present: mindfulness and its role in psychological well-being. Journal of Personality and Individual Differences.

[CR13] Brown KW, Ryan RM (2004). Perils and promise in defining and measuring mindfulness: observations from experience. Clinical Psychology: Science and Practice.

[CR14] Brown KW, Ryan RM, Creswell JD (2007). Mindfulness: theoretical foundations and evidence for its salutary effects. Psychological Inquiry.

[CR15] De Raedt R, Koster EHW (2010). Understanding vulnerability for depression from a cognitive neuroscience perspective: a reappraisal of attentional factors and a new conceptual framework. Cognitive, Affective & Behavioral Neuroscience.

[CR16] De Raedt, R., Baert, S., Demeijer, I., (…), Van Aalderen, J.R., & Speckens, A. (2012). Changes in attentional processing of emotional information following mindfulness-based cognitive therapy in people with a history of depression: towards an open attention for all emotional experiences. *Cognitive Therapy and Research*, 36, 612–620.

[CR17] Field, A. P. (2013). *Discovering statistics using IBM SPSS statistics: and sex and drugs and rock 'n' roll* (fourth ed.). London: Sage publications.

[CR18] Fledderus M, Oude Voshaar MAH, Ten Klooster PM, Bohlmeijer ET (2012). Further evaluation of the psychometric properties of the acceptance and action questionnaire-II. Psychological Assessment.

[CR19] Fletcher L, Hayes SC (2005). Relational frame theory, acceptance and commitment therapy, and a functional analytic definition of mindfulness. Journal of Rational-Emotive and Cognitive-Behavioral Therapy.

[CR20] Fox E, Russo R, Bowles R, Dutton K (2001). Do threatening stimuli draw or hold visual attention in subclinical anxiety?. Journal of Experimental Psychology: General.

[CR21] Garland EL, Howard MO (2013). Mindfulness-oriented recovery enhancement reduces pain attentional bias in chronic pain patients. Psychotherapy and Psychosomatics.

[CR22] Garland EL, Boettiger CA, Gaylord S, Chanon VW, Howard MO (2012). Mindfulness is inversely associated with alcohol attentional bias among recovering alcohol-dependent adults. Cognitive Therapy and Research.

[CR23] Gu J, Strauss C, Bond R, Cavanagh K (2015). How do mindfulness-based cognitive therapy and mindfulness-based stress reduction improve mental health and wellbeing? A systematic review and meta-analysis of mediation studies. Clinical Psychology Review.

[CR24] Hallion LS, Ruscio AM (2011). A meta-analysis of the effect of cognitive bias modification on anxiety and depression. Psychological Bulletin.

[CR25] Hanley A, Garland E, Canto A, Warner A, Hanley R, Dehili V, Proctor A (2015). Dispositional mindfulness and bias in self-theories. Mindfulness.

[CR26] Hayes, A.F. (2012). PROCESS [SPSS Macro]. Retrieved from http://afhayes.com/introduction-to-mediation-moderation-and-conditional-process-analysis.html

[CR27] Hayes SC, Strosahl KD, Wilson KG (1999). Acceptance and commitment therapy: an experiential approach to behavior change.

[CR28] Hayes, S. C., Strosahl, K. D., & Wilson, K. G. (2012). *Acceptance and commitment therapy: the process and practice of mindful change* (Second ed.). New York: Guilford Press.

[CR29] Hilton, L., Hempel, S., Ewing, B. A., Apaydin, E., Xenakis, L., Newberry, S., Calaiaco, B., Ruelaz Maher, A., Shanman, R. M., Sorbero, M. E., & Maglione, M. A. (2016). Mindfulness mediation for chronic pain: systematic review and meta-analysis. *Annals of Behavioral Medicine*, 1–15. 10.1007/s12160-016-9844-2 PMC5368208 27658913

[CR30] Hofmann SG, Sawyer AT, Witt AA, Oh D (2010). The effect of mindfulness-based therapy on anxiety and depression: a meta-analytic review. Journal of Consulting and Clinical Psychology.

[CR31] Jacobs N, Kleen M, De Groot F, A-Tjak J (2008). Het meten van experientiële vermijding. De Nederlandstalige versie van de Acceptance and Action Questionnaire-II (AAQ-II). Gedragstherapie.

[CR32] Kabat-Zinn J (1990). Full catastrophe living: using the wisdom of your body and mind to face stress, pain and illness.

[CR33] Kiken LG, Shook NJ (2011). Looking up: mindfulness increases positive judgments and reduces negativity bias. Social Psychology and Personality Science.

[CR34] Lakey CE, Kernis MH, Heppner WL, Lance CE (2008). Individual differences in authenticity and mindfulness as predictors of verbal defensiveness. Journal of Research in Personality.

[CR35] Lang TJ, Blackwell SE, Harmer CJ, Davison P, Holmes EA (2012). Cognitive bias modification using mental imagery for depression: developing a novel computerized intervention to change negative thinking styles. European Journal of Personality.

[CR36] Lemmer G, Gollwitzer M (2017). The “true” indirect effect won’t (always) stand up: when and why reverse mediation testing fails. Journal of Experimental Social Psychology.

[CR37] Li W, Howard MO, Garland EL, McGovern P, Lazar M (2017). Mindfulness treatment for substance misuse: a systematic review and meta-analysis. Journal of Substance Abuse Treatment.

[CR38] Mackintosh B, Mathews A, Yiend J, Ridgeway V, Cook E (2006). Induced biases in emotional interpretation influence stress vulnerability and endure despite changes in context. Behavior Therapy.

[CR39] MacLeod C, Cohen IL (1993). Anxiety and the interpretation of ambiguity: a text comprehension study. Journal of Abnormal Psychology.

[CR40] Mathews A, Mackintosh B (2000). Induced emotional interpretation bias and anxiety. Journal of Abnormal Psychology.

[CR41] Mayer B, Muris P, Busser K, Bergamin J (2009). A disgust mood state causes a negative interpretation bias, but not in the specific domain of body-related concerns. Behaviour Research and Therapy.

[CR42] Pepping CA, Duvenage M, Cronin TJ, Lyons A (2016). Adolescent mindfulness and psychopathology: the role of emotion regulation. Personality and Individual Differences.

[CR43] Qualtrics, Provo, UT, USA (n.d.), http://www.qualtrics.com.

[CR44] Schmertz SK, Masuda A, Anderson PL (2012). Cognitive processes mediate the relation between mindfulness and social anxiety within a clinical sample. Journal of Clinical Psychology.

[CR45] Segal ZV, Williams JMG, Teasdale JT (2001). Mindfulness-based cognitive therapy for depression: a new approach to preventing relapse.

[CR46] Shorey RC, Brasfield H, Anderson S, Stuart GL (2014). Differences in trait mindfulness across mental health symptoms among adults in substance use treatment. Substance Use and Misuse.

[CR47] Spielberger C, Gorsuch R, Lushene R (1983). Manual for the state-trait anxiety inventory.

[CR48] Thoemmes F (2015). Reversing arrows in mediation models does not distinguish plausible models. Basic and Applied Social Psychology.

[CR49] Thompson BL, Waltz J (2007). Everyday mindfulness and mindfulness meditation: overlapping constructs or not?. Personality and Individual Differences.

[CR50] Vago DR (2014). Mapping modalities of self-awareness in mindfulness practice: a potential mechanism for clarifying habits of mind. Annals of the New York Academy of Sciences.

[CR51] Van der Does AJW (2002). Handleiding bij de Nederlandstalige versie van Beck depression inventory—second edition (BDI-II-NL).

[CR52] Van der Ploeg HM, Defares PB, Spielberger CD (1980). Handleiding bij de Zelfbeoordelingsvragenlijst, ZBV. Een Nederlandse bewerking van de Spielberger State-Trait Anxiety Inventory, STAI-DY.

